# Abdominal Adipose Tissue was Associated with Glomerular Hyperfiltration among Non- Diabetic and Normotensive Adults with a Normal Body Mass Index

**DOI:** 10.1371/journal.pone.0141364

**Published:** 2015-10-23

**Authors:** Jeonghwan Lee, Hye Jin Kim, Belong Cho, Jin Ho Park, Ho Chun Choi, Cheol Min Lee, Seung Won Oh, Hyuktae Kwon, Nam Ju Heo

**Affiliations:** 1 Department of Internal Medicine, Hallym University Hangang Sacred Heart Hospital, Seoul, Korea; 2 Department of Family Medicine, Seoul National University Hospital, Seoul National University College of Medicine, Seoul, South Korea; 3 Department of Family Medicine, Healthcare System Gangnam Center of Seoul National University Hospital, Seoul, South Korea; 4 Subdivision of Nephrology, Department of Internal Medicine, Healthcare System Gangnam Center, Seoul National University Hospital, Seoul, South Korea; University of Leipzig, GERMANY

## Abstract

Glomerular hyperfiltration is recognized as an early marker of progressive kidney dysfunction in the obese population. This study aimed to identify the relationship between glomerular hyperfiltration and body fat distribution measured by computed tomography (CT) in healthy Korean adults. The study population included individuals aged 20–64 years who went a routine health check-up including an abdominal CT scan. We selected 4,378 individuals without diabetes and hypertension. Glomerular filtration rate was estimated using the CKD-EPI equation, and glomerular hyperfiltration was defined as the highest quintile of glomerular filtration rate. Abdominal adipose tissue areas were measured at the level of the umbilicus using a 16-detector CT scanner, and the cross-sectional area was calculated using Rapidia 2.8 CT software. The prevalence of glomerular hyperfiltration increased significantly according to the subcutaneous adipose tissue area in men (OR = 1.74 (1.16–2.61), P for trend 0.016, for the comparisons of lowest vs. highest quartile) and visceral adipose tissue area in women (OR = 2.34 (1.46–3.75), P for trend < 0.001) in multivariate analysis. After stratification by body mass index (normal < 23 kg/m^2^, overweight ≥ 23 kg/m^2^), male subjects with greater subcutaneous adipose tissue, even those in the normal BMI group, had a higher prevalence of glomerular hyperfiltration (OR = 2.11 (1.17–3.80), P for trend = 0.009). Among women, the significance of visceral adipose tissue area on glomerular hyperfiltration resulted from the normal BMI group (OR = 2.14 (1.31–3.49), P for trend = 0.002). After menopause, the odds ratio of the association of glomerular hyperfiltration with subcutaneous abdominal adipose tissue increased (OR = 2.96 (1.21–7.25), P for trend = 0.013). Subcutaneous adipose tissue areas and visceral adipose tissue areas are positively associated with glomerular hyperfiltration in healthy Korean adult men and women, respectively. In post-menopausal women, visceral adipose tissue area shows significant positive association with glomerular hyperfiltration as in men.

## Introduction

Obesity is recognized as an independent risk factor for the development of chronic kidney disease and end-stage renal disease [1]. The risk of end-stage renal disease associated with obesity is almost comparable to that of hypertension [2]. Considering the increasing prevalence of obesity, renal disease related to obesity is also expected to grow. Thus, early detection of obesity-related kidney disease and proper management of obesity are needed among the healthy population.

Previous animal and human studies have shown that obesity is closely related with renal hemodynamic changes, including increases in the glomerular filtration rate (GFR) and renal plasma flow [3–6]. In addition, obesity-related glomerular hyperfiltration is ameliorated after weight loss [7]. In the pathogenesis of obesity-related kidney disease, glomerular hyperfiltration can be associated with enhanced intraglomerular pressure transmission and subsequent structural abnormality of glomerulomegaly, which is a histological hallmark of obesity-related glomerulopathy [8–10]. Glomerular hyperfiltration, which is observed before the appearance of glomerulomegaly or renal dysfunction, could be an early marker of obesity-related kidney disease, similar to the glomerular hyperfiltration that precedes the development of microalbuminuria in patients with hypertension or diabetes [11–13].

Although overall obesity is generally evaluated by body mass index, this index is a poor indicator of body fat distribution [14]. Central obesity is thought to be more accurate measurement for the determination of adverse clinical outcomes compared with general obesity represented by body mass index [15,16]. Central obesity is an independent risk factor for microalbuminuria and renal dysfunction [17,18]. Recently, the distribution of abdominal adipose tissue, which is not easily measured by body mass index or waist circumference, was revealed as an important factor related to cardiometabolic risk [19–21]. Therefore, specific abdominal adiposity, as measured by CT scan, might be a better predictor of chronic kidney disease compared with traditional clinical anthropometric measures such as body mass index, waist circumference or waist-hip ratio.

There are several reports examining the relationship between obesity and renal hemodynamic changes [3,5,6,8,22–26]. However, the relationship between abdominal fat distribution and glomerular hyperfiltration and has not been studied to date. In addition, despite the significant influences of diabetes, hypertension, and obesity on glomerular hyperfiltration, few studies have adjusted for these factors. In this study, we aimed to analyze the associations between glomerular hyperfiltration and abdominal fat distribution using CT scan in non-diabetic, non-hypertensive Korean adults.

## Materials and Methods

### Study Participants

We conducted a cross-sectional observational study of 7,978 subjects between 20 and 64 years old who underwent routine comprehensive health check-ups, including abdominal CT scan, at Seoul National University Hospital Healthcare System Gangnam Center from March 2008 through February 2010. The exclusion criteria were as follows: renal function impairment or evidence of chronic kidney disease (defined as having estimated GFR by CKD-EPI equation < 60 ml/min/1.73 m^2^), diabetes, hypertension, or any type of cancer [27]. Thus, 5,042 subjects were eligible for this study.

Most subjects either voluntarily paid for their health check-ups if they were in need of medical attention or were financially supported by their company. The Institutional Review Board of Seoul National University Hospital approved this study (number: H-1003-027-311), and written informed consent was obtained from all participants.

### Clinical and Laboratory Assessments

All subjects completed a medical questionnaire, including past medical history, smoking, alcohol consumption, hormone replacement therapy, and menopause status. Current smoking was defined as smoking at least one cigarette per day for the previous 12 months. Height, weight, waist circumference, and blood pressure were measured directly by trained nurses. Blood samples were drawn for the measurement of serum glucose, triglyceride, HDL cholesterol, and insulin levels in the morning after participants had fasted for at least 12 hours. All biochemical determinations were performed in the same laboratory using standard laboratory methods. Triglyceride was log-transformed to ensure a normal distribution. Body mass index was calculated as the weight in kilograms divided by the square of the height in meters (kg/m^2^). Insulin resistance was estimated using the homeostasis model assessment (HOMA) index: HOMA-IR = fasting plasma insulin (μU/L) × fasting plasma glucose (mmol/L) / 22.5 [28].

### Renal Function Measurements

Recent guidelines recommend using serum creatinine and a GFR estimating equation for the measurement of renal function [29]. In this study, GFR was estimated using the CKD-EPI creatinine equation: 141 × min(SCr/κ, 1)^α^ × max(SCr/κ, 1)^-1.209^ × 0.993^Age^ [× 1.018 if female] [× 1.159 if black], where SCr is serum creatinine (in mg/dl), κ is 0.7 for females and 0.9 for males, α is -0.329 for females and -0.411 for males, min is the minimum of SCr/κ or 1, and max is the maximum of SCr/κ or 1 [30]. Because a clear standard glomerular hyperfiltration has not been established, this study defined glomerular hyperfiltration as the highest quintile after stratifying by gender (men 93.8 ml/min/1.73 m^2^, women 98.9 ml/min/1.73 m^2^), as described in a recent study [31].

### Measurement of Abdominal Adipose Tissue Areas by CT Scan

The adipose tissue areas were measured at the level of the umbilicus using a 16-detector row CT scanner (Somatom Sensation 16, Siemens Medical Solutions, Forchheim, Germany), as previously described [32,33]. In brief, a 5-mm-thick umbilical-level abdominal section was obtained. The cross-sectional area (cm^2^) of the abdominal fat was calculated using Rapidia 2.8 CT software (INFINITT, Seoul, Korea). The visceral adipose tissue area was defined as intra-peritoneal fat bound by parietal peritoneum or transversalis fascia, and the subcutaneous adipose tissue area was defined as fat areas external to the abdomen and back muscles. The total adipose tissue area was calculated based on the summation of visceral and subcutaneous adipose tissue areas. Because a clear standard for normal abdominal fat has not been established, we used the lowest quartile as the reference group after subdividing abdominal fat amounts by quartile.

### Statistical Analysis

All analyses were performed after stratification by gender. In the descriptive analysis of the demographic and clinical characteristics, continuous variables were expressed as the mean and standard deviation, and categorical variables were described numerically with a percentage. Correlation between adipose tissue area and GFR were analyzed by simple linear regression methods. We compared the clinical characteristics of the participants in the glomerular hyperfiltration group and the non-glomerular hyperfiltration group using a t-test for continuous variables and a chi-square test for categorical variables. Univariate and multivariate logistic regression analysis were performed to assess the effect of abdominal adipose tissue area on glomerular hyperfiltration. In multivariate analysis, variables (age, height, weight, waist circumference, systolic blood pressure, diastolic blood pressure, log value of triglyceride, total cholesterol, LDL cholesterol, HDL cholesterol, HbA1c, glucose level, HOMA-IR, body surface are, smoking status, and status of menopause) were selected by the stepwise backward methods including variables of P value below 0.05 and excluding variables of P value above 0.10. Covariates were excluded after interactions between significant covariates were tested. The cubic spline curve was adjusted for the aforementioned confounding variables and was fit to further characterize the nature of the relationship between abdominal adiposity distribution and glomerular hyperfiltration. The data was expressed as odds ratios (ORs), 95% confidence intervals, and P for trend. All analyses were performed using IBM SPSS version 21.0 (IBM Corp, New York, USA) except adjusted cubic splines that was tested with R 2.15.2 and rms packages. A two-tailed P value below 0.05 was considered statistically significant.

## Results

### Characteristics of Study Subjects

**Fig 1:**
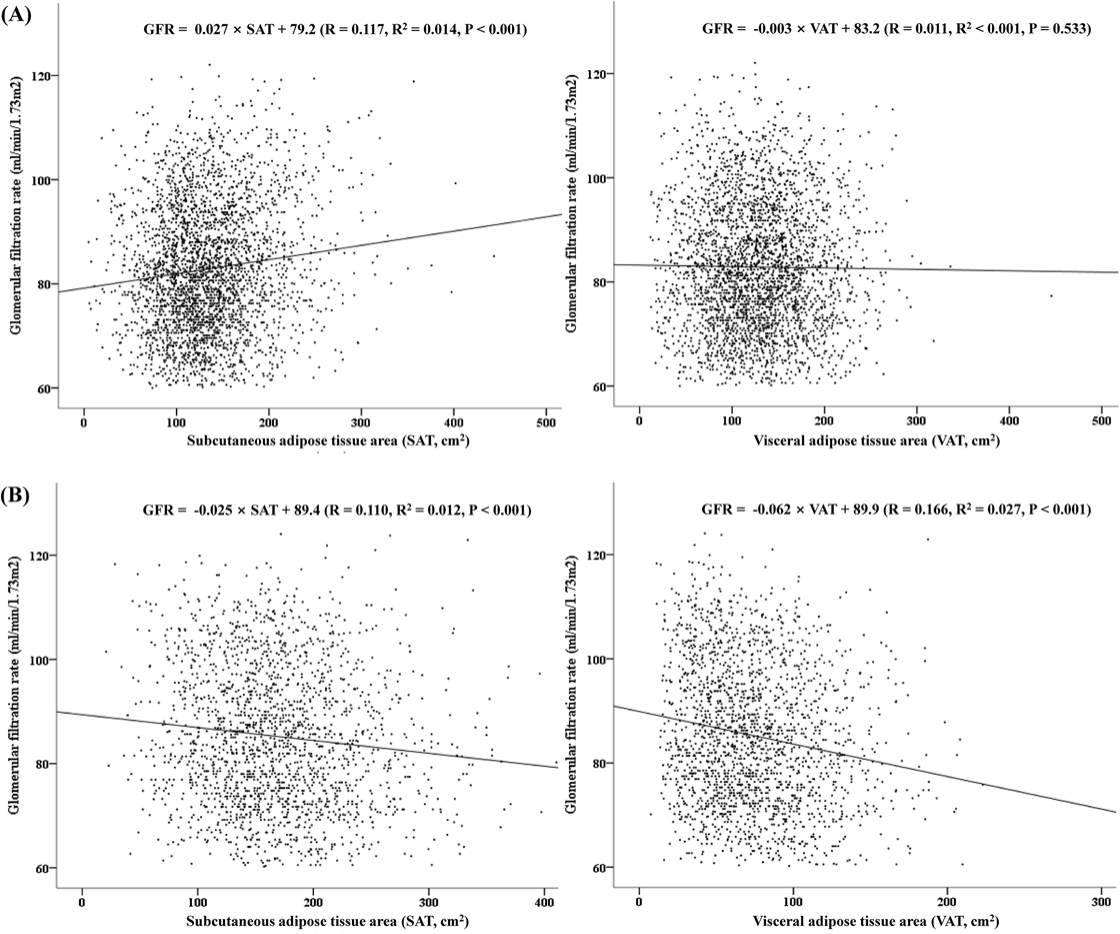
Correlation between glomerular filtration rate and subcutaneous and visceral adipose tissue area. Glomerular filtration rate was positively correlated with subcutaneous adipose tissue area (1-A: SAT, P < 0.001, R^2^ = 0.014) in men. Visceral adipose tissue area did not show correlation with glomerular filtration rate (VAT, P = 0.533, R^2^ < 0.001). In women, glomerular filtration rate was negatively correlated with both visceral adipose tissue area (Fig 1-B; VAT, P < 0.001, R^2^ = 0.027) and subcutaneous adipose tissue area (SAT, P < 0.001, R^2^ = 0.012).

Among the 5,042 subjects, there were 3,046 (60.4%) men. The mean age was 47.7 ± 8.2 years in men and 48.6 ± 7.9 years in women. The mean body mass index was 24.3 ± 2.6 in men and 21.9 ± 2.6 in women. The mean visceral and subcutaneous adipose tissue areas were 128.0 ± 50.8 cm^2^ and 134.6 ± 51.6 cm^2^ in men and 74.3 ± 35.7 cm^2^ and 168.2 ± 59.8 cm^2^ in women, respectively. The mean GFR was 82.9 ± 12.1 ml/min/1.73 m^2^ in men and 85.2 ± 13.5 ml/min/1.73 m^2^ in women. Fig 1 illustrates the relationship between the adipose tissue area and glomerular filtration rate. In men, GFR was positively correlated with subcutaneous adipose tissue area (Fig 1A; P < 0.001, R^2^ = 0.014), and the association of subcutaneous adipose tissue with GFR was stronger than that of visceral adipose tissue area (R^2^ = 0.001). In women, glomerular filtration rate was negatively correlated with both visceral and subcutaneous adipose tissue area, and the association was stronger for the visceral adipose tissue area (Fig 1B; P < 0.001, R^2^ = 0.027) than for the subcutaneous adipose tissue area (R^2^ = 0.012).

Table 1 shows the general characteristics of the glomerular hyperfiltration group and the non-glomerular hyperfiltration group. The age in the glomerular hyperfiltration group was lower in both men and women. In men, the prevalence of current smoker was higher, and the level of HbA1c and diastolic blood pressure were lower in the glomerular hyperfiltration group. Subcutaneous adipose tissue area in the glomerular hyperfiltration group was larger than that in the normal glomerular filtration group. In women, body mass index, waist circumference, glucose, HbA1c, LDL cholesterol level, HOMA-IR, systolic and diastolic blood pressure, and the prevalence of menopause were lower in the glomerular hyperfiltration group. Subcutaneous and visceral adipose tissue area were smaller among women with the glomerular hyperfiltration than those in the normal glomerular filtration group.

**Table 1:** Patient characteristics according to the glomerular filtration rate.

	Men (N = 3,046)			Women (N = 1,996)		
	Normal glomerular filtration group (GFR ≤ 93.8 ml/min/1.73m^2^)	Glomerular hyperfiltration group (GFR > 93.8 ml/min/1.73m^2^)	P	Normal glomerular filtration group (GFR ≤ 98.0 ml/min/1.73m^2^)	Glomerular hyperfiltration group (GFR > 98.0 ml/min/1.73m^2^)	P
	N = 2,431	N = 615		N = 1,596	N = 400	
**Age, years**	48.7(8.0)	44.1(8.3)	<0.001	49.6(7.8)	44.8(7.3)	<0.001
**Current smoker**	848(35.9)	249(41.5)	0.011	79(5.3)	27(7.0)	0.175
**Postmenopause**				752 (47.1)	85 (21.3)	<0.001
**Measurements of obesity**						
** BMI, kg/m** ^**2**^	24.3(2.5)	24.4(2.9)	0.727	22.1(2.6)	21.2(2.5)	<0.001
** Waist circumference, cm**	71.1(8.9)	71.7(9.9)	0.162	80.3(7.2)	77.3(6.8)	<0.001
** CT measurements**						
** TAT, cm** ^**2**^	260.5(88.7)	270.9(95.6)	0.015	247.7(86.9)	221.5(79.6)	<0.001
** VAT, cm** ^**2**^	128.3(50.9)	126.5(50.4)	0.438	76.5(36.0)	65.6(33.3)	<0.001
** SAT, cm** ^**2**^	132.1(49.7)	144.3(57.7)	<0.001	171.2(60.4)	155.9(55.7)	<0.001
**Other metabolic risk factors**						
** Fasting glucose, mg/dl**	93.0(10.1)	92.8(9.9)	0.706	87.9(10.0)	86.6(9.9)	0.022
** HbA1c (%)**	5.70(0.28)	5.65(0.25)	<0.001	5.76(0.27)	5.67(0.25)	<0.001
** Triglyceride, mg/dl**	123.3(74.2)	123.1(75.1)	0.958	83.4(42.1)	72.0(36.9)	<0.001
** HDL, mg/dl**	50.3(11.7)	50.4(11.1)	0.852	60.1(13.6)	61.3(13.1)	0.013
** LDL, mg/dl**	127.7(31.1)	125.9(30.8)	0.185	121.4(31.5)	115.2(29.4)	<0.001
** HOMA-IR**	1.8(0.9)	1.8(1.0)	0.658	1.6(0.8)	1.4(0.8)	0.002
** SBP, mmHg**	114.6(10.7)	115.0(10.8)	0.387	109.2(12.6)	106.3(12.5)	<0.001
** DBP, mmHg**	74.8(8.3)	73.9(8.3)	0.013	68.4(9.3)	66.0(9.4)	<0.001

Results are expressed as frequencies (percentage) and mean values (standard deviation) as appropriate. Glomerular hyperfiltration was defined as the highest quintile of creatinine clearance.

BMI, body mass index; CT, computed tomography; DBP, diastolic blood pressure; HDL, high density lipoprotein levels; HOMA-IR, homeostatic model assessment of insulin resistance; SAT, subcutaneous adipose tissue area; SBP, systolic blood pressure; TAT, total adipose tissue area; VAT, visceral adipose tissue area

### Glomerular Hyperfiltration and the Distribution of Abdominal Adipose Tissue

The adjusted odds ratios of glomerular hyperfiltration in relation to abdominal adipose tissue area stratified by sex are presented in Table 2. Among men, the prevalence of glomerular hyperfiltration increased according to increases in the subcutaneous adipose tissue area (OR = 1.74 (1.16–2.61) for comparisons of lowest vs. highest quartile; P for trend = 0.016) in multivariate analysis. However, visceral adipose tissue area was not associated with glomerular hyperfiltration (OR = 1.13 (0.79–1.64) for comparisons of lowest vs. highest quartile; P for trend = 0.475). In women, the prevalence of glomerular hyperfiltration increased in accordance with increases in both subcutaneous (OR = 1.79 (1.12–2.88) for comparisons of lowest vs. highest quartile; P for trend = 0.010) and visceral (OR = 2.34 (1.46–3.75) for comparisons of lowest vs. highest quartile; P for trend < 0.001) adipose tissue. The odds ratios of visceral adipose tissue area for the glomerular hyperfiltration were higher than those of subcutaneous adipose tissue area.

**Table 2:** The prevalence of glomerular hyperfiltration in relation to the area of abdominal adipose tissue.

	Men (N = 3,046)			Women (N = 1,996)		
	N	OR (95% CI)	P	N	OR (95% CI)	P
**VAT**			0.475			<0.001^a^
**Q1**	761	1		488	1	
**Q2**	762	1.14(0.86–1.52)	0.369	489	1.20(0.86–1.67)	0.278
**Q3**	762	1.20(0.88–1.66)	0.254	495	1.79(1.22–2.62)	0.003
**Q4**	761	1.13(0.79–1.64)	0.505	494	2.34(1.46–3.75)	<0.001
**SAT**			0.016^a^			0.010^a^
**Q1**	761	1		484	1	
**Q2**	762	1.29(0.94–1,75)	0.112	493	1.18(0.84–1.65)	0.335
**Q3**	762	1.19(0.84–1.68)	0.330	494	1.48(1.02–2.15)	0.041
**Q4**	761	1.74(1.16–2.61)	0.007	495	1.79(1.12–2.88)	0.016

Q1~Q4, quartile group of each adipose tissue; SAT, subcutaneous adipose tissue area; VAT, visceral adipose tissue area

Range of quartile in men: VAT Q1, (~92.3), Q2, (92.3~126.8), Q3, (126.8~160.3), Q4, (160.3~) SAT Q1, (~99.9), Q2, (99.9~129.0), Q3, (129.0~161.5), Q4, (161.5~)

Range of quartile in women: VAT Q1, (~47.1), Q2: (47.1~69.7), Q3: (69.7~96.3), Q4: (96.3~) SAT Q1, (~,128.4), Q2, (128.4~162.6), Q3, (162.6~203.6), Q4, (203.6~)

^a^ P for trend

Multivariate analysis was adjusted for age, height, weight, waist circumference, systolic blood pressure, diastolic blood pressure, triglyceride(log), HbA1c, glucose, and body surface area in SAT and VAT of men; age, waist circumference, diastolic blood pressure, triglyceride(log), HbA1c, HOMA-IR, and status of menopause in SAT and VAT of women.

**Fig 2:**
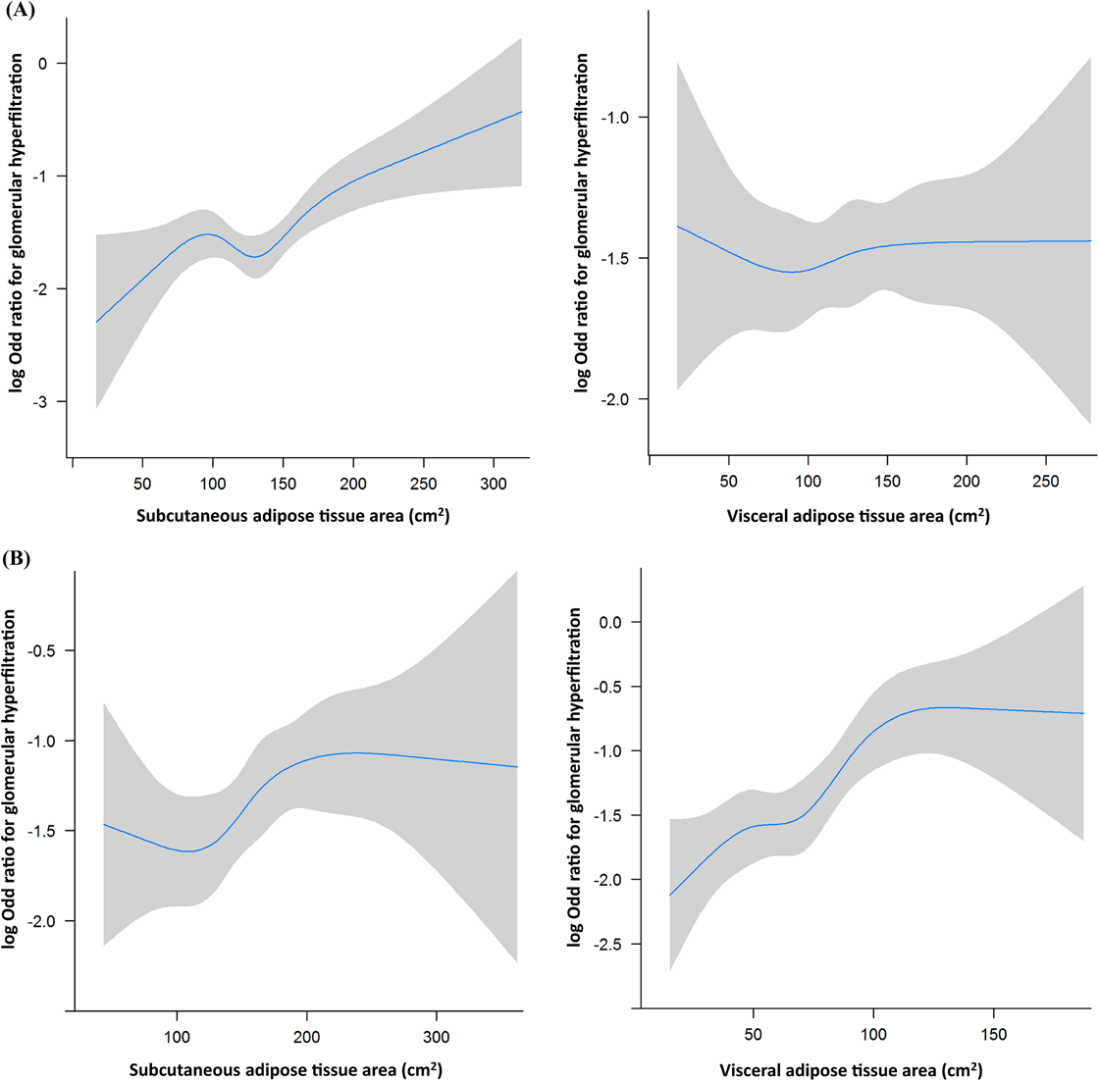
Adjusted cubic splines for glomerular hyperfiltration in men and women according to abdominal adipose tissue area. The log value of odds ratio for glomerular hyperfiltration increased linearly according to the increase of subcutaneous adipose tissue area among men (2-A). In women, visceral adipose tissue area showed stronger correlation to glomerular hyperfiltration than subcutaneous adipose tissue area (2-B).

Fig 2 illustrates the adjusted cubic splines for glomerular hyperfiltration among men and women according to abdominal adipose tissue area. The log value of odds ratio for glomerular hyperfiltration increased linearly according to the increase of subcutaneous adipose tissue area among men (Fig 2A). However, the log value of odds ratio did not increased according to the visceral adipose tissue area. In women, visceral adipose tissue area showed stronger linear correlation to glomerular hyperfiltration than subcutaneous adipose tissue area (Fig 2B).

### The Relationship between Glomerular Hyperfiltration and Abdominal Adipose Tissue Stratified by Body Mass Index

The prevalence of glomerular hyperfiltration was analyzed stratified by body mass index (normal BMI < 23 kg/m^2^, overweight ≥ 23 kg/m^2^). Men with greater subcutaneous adipose tissue areas had a higher prevalence of glomerular hyperfiltration with a linear trend; this was also the case for subjects with a normal body mass index (SAT, OR = 2.11 (1.17–3.80) for comparisons of lowest vs. highest quartile, P for trend = 0.009) (Table 3). On the contrary, the visceral adipose tissue area was not associated with glomerular hyperfiltration in the men with overweight group (BMI ≥ 23 kg/m^2^, VAT: OR = 0.88 (0.60–1.30) for comparisons of lowest vs. highest quartile, P for trend = 0.655).

**Table 3:** The relationship between glomerular hyperfiltration and the distribution of abdominal adipose tissue according to body mass index.

	Men (N = 3,046)						Women (N = 1,996)					
	Normal BMI group (BMI < 23 kg/m^2^, N = 891)			Overweight group (BMI ≥ 23 kg/m^2^, N = 2,155)			Normal BMI group (BMI < 23 kg/m^2^, N = 1,394)			Overweight group (BMI ≥ 23 kg/m^2^, N = 602)		
	N	OR (95% CI)	P	N	OR (95% CI)	P	N	OR (95% CI)	P	N	OR (95% CI)	P
**VAT**			0.037^a^			0.655^a^			0.002^a^			0.224^a^
**Q1**	223	1		538	1		340	1		148	1	
**Q2**	222	1.28(0.79–2.09)	0.319	539	0.80(0.58–1.12)	0.195	341	1.00(0.68–1.45)	0.989	151	2.30(1.17–4.52)	0.016
**Q3**	223	1.01(0.60–1.71)	0.966	540	0.88(0.63–1.25)	0.482	343	1.22(0.80–1.88)	0.356	147	2.48(1.20–5.12)	0.014
**Q4**	223	1.98(1.16–3.39)	0.012	538	0.88(0.60–1.30)	0.523	347	2.14(1.31–3.49)	0.003	149	1.72(0.72–4.13)	0.225
**SAT**			0.009^a^			0.030^a^			0.149^a^			0.496^a^
**Q1**	223	1		539	1		341	1		149	1	
**Q2**	222	1.70(1.00–2.88)	0.050	538	0.84(0.59–1.20)	0.342	339	0.93(0.63–1.39)	0.725	149	1.05(0.53–2.07)	0.894
**Q3**	223	2.15(1.23–3.74)	0.007	540	0.97(0.68–1.38)	0.853	345	1.42(0.94–2.14)	0.093	150	1.88(0.96–3.69)	0.066
**Q4**	223	2.11(1.17–3.80)	0.013	538	1.57(1.03–2.37)	0.035	346	1.28(0.78–2.10)	0.332	147	1.13(0.48–2.64)	0.786

Q1~Q4, quartile group of each adipose tissue; BMI, body mass index; SAT, subcutaneous adipose tissue area; VAT, visceral adipose tissue area; Q1~Q4, quartile group of each adipose tissue

Range of quartile in men (normal BMI): VAT Q1, (~60.0), Q2, (60.0~86.8), Q3, (86.8~116.2), Q4, (116.2~) SAT Q1, (~72.6), Q2, (72.6~94.0), Q3, (94.0~114.2), Q4, (114.2~)

Range of quartile in women (normal BMI): VAT Q1, (~40.6), Q2: (40.6~57.6), Q3: (57.6~78.3), Q4: (78.3~) SAT Q1, (~114.7), Q2, (114.7~145.8), Q3, (145.8~176.0), Q4, (176.2~)

Range of quartile in men (overweight): VAT Q1, (~110.5), Q2, (110.5~140.7), Q3, (140.7~171.9), Q4, (171.9~) SAT Q1, (~119.2), Q2, (119.2~144.1), Q3, (144.1~175.5), Q4, (175.5~)

Range of quartile in women (overweight): VAT Q1, (~78.7), Q2: (78.7~100.2), Q3: (100.2~123.6), Q4: (123.6~) SAT Q1, (~177.7), Q2, (177.7~213.1), Q3, (213.1~252.6), Q4, (252.6~)

^a^ P for trend

Multivariate analysis was adjusted for age, body mass index, weight, systolic blood pressure, diastolic blood pressure, and body surface area in the group of men with normal BMI (< 23); age, waist circumference, triglyceride(log), HbA1c and glucose in the group of men with overweight BMI (≥ 23); age, waist circumference, diastolic blood pressure, triglyceride(log), HbA1c, glucose, HOMA-IR, and status of menopause in the group of women.

Among women, increase in subcutaneous adipose tissue area was not associated with glomerular hyperfiltration in both normal body mass index group and overweight group. However, visceral adipose tissue area was significantly associated with glomerular hyperfiltration in the subjects with a normal body mass index (OR = 2.14 (1.31–3.49) for comparisons of lowest vs. highest quartile, P for trend = 0.002).

### The Relationship between Glomerular Hyperfiltration and Abdominal Adipose Tissue according to Menopausal Status

The relationship between glomerular hyperfiltration and abdominal adipose tissue was compared according to menopausal status (Table 4). Increase in visceral adipose tissue area, but not in subcutaneous adipose tissue area, was associated with a greater prevalence of glomerular hyperfiltration in premenopausal women (VAT: OR = 1.85 (1.10–3.10), P for trend = 0.020; SAT: OR = 1.36 (0.80–2.30) for comparisons of lowest vs. highest quartile, P for trend = 0.016). However, after menopause, increases of both subcutaneous and visceral abdominal adipose tissue area were significantly associated with glomerular hyperfiltration (VAT: OR = 2.45 (1.01–5.98), for comparisons of lowest vs. highest quartile, P for trend = 0.006; SAT: OR = 2.96 (1.21–7.25), for comparisons of lowest vs. highest quartile, P for trend = 0.013).

**Table 4:** The relationship between glomerular hyperfiltration and the distribution of abdominal adipose tissue according to menopause status in women (N = 1,996).

	Premenopause (N = 1,159)			Postmenopause (N = 837)		
	N	OR (95% CI)	P	N	OR (95% CI)	P
**VAT**			0.020^a^			0.006^a^
**Q1**	282	1		209	1	
**Q2**	284	1.11(0.75–1.63)	0.531	209	0.93(0.43–1.98)	0.843
**Q3**	283	1.27(0.82–1.96)	0.301	210	3.58(1.69–7.59)	0.001
**Q4**	288	1.85(1.10–3.10)	0.021	209	2.45(1.01–5.98)	0.001
**SAT**			0.160^a^			0.013^a^
**Q1**	282	1		209	1	
**Q2**	284	1.02(0.68–1.51)	0.938	210	1.54(0.77–3.08)	0.221
**Q3**	283	1.38(0.90–2.11)	0.144	209	2.29(1.06–4.93)	0.034
**Q4**	288	1.36(0.80–2.30)	0.261	209	2.96(1.21–7.25)	0.017

Q1~Q4, quartile group of each adipose tissue; SAT, subcutaneous adipose tissue area; VAT, visceral adipose tissue area; Q1~Q4, quartile group of each adipose tissue

Range of quartile in men (premenopause): VAT Q1, (~41.0), Q2, (41.0~58.6), Q3, (58.6~82.2), Q4, (82.2~) SAT Q1, (~117.8), Q2, (117.8~151.9), Q3, (151.9~187.2), Q4, (187.2~)

Range of quartile in women (postmenopause): VAT Q1, (~62.3), Q2: (62.3~84.7), Q3: (84.7~111.3), Q4: (111.3~) SAT Q1, (~142.2), Q2, (142.2~178.3), Q3, (178.3~219.6), Q4, (219.6~)

Multivariate analysis was adjusted for age, waist circumference, diastolic blood pressure, triglyceride(log), HbA1c, and HOMA-IR in premenopause women; age and waist circumference in postmenopause women.

^a^ P for trend

## Discussion

In this study, we investigated the relationship between abdominal fat distribution and glomerular hyperfiltration in healthy, normotensive, non-diabetic Korean adults. Our results demonstrate that subcutaneous and visceral abdominal adipose tissue area in men and women, respectively, are independent risk factors for glomerular hyperfiltration. Subcutaneous abdominal adipose tissue area was independently associated with glomerular hyperfiltration among men, even in participants with a normal body mass index. Visceral adipose tissue area was associated with glomerular hyperfiltration only in the normal body mass index group. However, in women, the association between subcutaneous adipose tissue and glomerular hyperfiltration trended toward being dependent on the effect of body mass index. On the contrary, visceral adipose tissue area showed significant association with glomerular hyperfiltration even in the normal body mass group. Interestingly, after menopause, the association of subcutaneous adipose tissue area with glomerular hyperfiltration increased significantly.

To the best of our knowledge, this study is the first large-scale investigation reporting that abdominal fat distribution is positively associated with glomerular hyperfiltration. Several previous studies have explored the relationship between generalized or central obesity and glomerular hyperfiltration [6,22,23,34]. However, because most of these previous studies included a small number of obese patients in a case-control setting [5,7], these findings are difficult to apply to healthy, non-obese individuals. Other studies used body mass index or waist-hip ratio to define obesity; therefore, the effect of body fat distribution could not be assessed [6,22,23]. Lee et al. used CT scan to categorize female patients into visceral or subcutaneous abdominal fat-dominant groups [34]. Because we included 5,042, normotensive, non-diabetic, healthy men and women, the results of our study can be applied to the general population. In addition, we categorized central obesity by abdominal fat distribution. The metabolic effects of visceral and subcutaneous adipose tissue on glomerular hyperfiltration can also be differentiated in the results of this study.

Many previous studies reported that glomerular hyperfiltration could be an early indicator of renal dysfunction in diabetes [35–39], polycystic kidney disease [40], and focal segmental glomerulosclerosis [41]. An experimental study targeting rats showed that obese rats exhibited glomerular hyperfiltration and, eventually, showed focal segmental glomerulosclerosis [42]. A recent study suggested that the glomerular hyperfiltration observed in obese subjects might play a role in the pathogenesis of hypertension by enhancing sodium reabsorption and by mediating glomerular damage [24]. Several studies have suggested that microalbuminuria and changes in glomerular permeability might be related to altered renal hemodynamics and glomerular hyperfiltration [43,44]. In this study, we could not analyze the relationship between fat distribution and indicator of kidney injury, such as microalbuminuria. However, based on previous reports, we can estimate that glomerular hyperfiltration precedes the development of various kidney diseases and has an effect on the pathogenesis of kidney disease. Therefore, glomerular hyperfiltration can be used as a surrogate marker of progressive renal disease.

Obesity, measured by body mass index, is known to be independently associated with glomerular hyperfiltration, even in patients with hypertension or diabetes [24,25]. Increased adipose tissue can cause renal mesangial and epithelial cell injury and may promote kidney disease progression [45]. Chagnac et al. showed that the increase in GFR in severely obese patients can be partially corrected by weight loss after gastroplasty [7]. In particular, central obesity is independently associated with glomerular hyperfiltration [46]. Our finding that abdominal adipose tissue, especially the area of subcutaneous adipose tissue in men and visceral adipose tissue in women, is associated with glomerular hyperfiltration supports the hypothesis that central adiposity might be a risk factor for progressive renal insufficiency.

Conventionally, visceral adipose tissue area has been recognized as pathogenic fat deposition, and this area is more strongly associated with metabolic risk factors than the subcutaneous adipose tissue area [47]. Visceral adipose tissue is a key regulator of numerous adipokines and cytokines, including angiotensin [48,49]. In the present study, however, subcutaneous adipose tissue and visceral adipose tissue were associated with glomerular hyperfiltration. The association of glomerular hyperfiltration with abdominal adiposity was not proved in visceral adipose tissue area in men, but in subcutaneous adipose tissue area. In addition, subcutaneous adipose tissue area showed increased association with glomerular hyperfiltration after menopause. Although we did not investigate the metabolic effect of mediators associated with subcutaneous adipose tissue, there are several explanations for this strong association between subcutaneous adipose tissue and glomerular hyperfiltration. Leptin, which is released preferentially in subcutaneous adipose tissue versus visceral adipose tissue, stimulates renal sympathetic activity, eventually resulting in hypertension and glomerular hyperfiltration [25]. The effect of leptin, which is generally attenuated in obesity, is preserved in the renal sympathetic nervous system [50]. In addition, leptin can cross the blood-brain barrier and bind to receptors in the central nervous system. This stimulates the central sympathetic nervous system, which, in turn, leads to renin release from the kidney [51]. In healthy participants, leptin levels were closely associated with creatinine clearance and glomerular hyperfiltration [52]. In future studies, leptin levels should be measured to investigate the cause of glomerular hyperfiltration according to the distribution of abdominal adipose tissue.

This study has several limitations. Although we investigated the association between abdominal adipose tissue and glomerular hyperfiltration, the effects of glomerular hyperfiltration on kidney damage or renal dysfunction were not proved. Glomerular hyperfiltration itself is not an indicator or kidney injury or a manifestation of kidney disease. Glomerular hyperfiltration can occur, in physiological responses, as a result of a coordinated increase in glomerular capillary filtration surface area and increase in the single-nephron plasma flow without a pathogenic increase in glomerular capillary pressure [8,53]. In addition, bioactive molecules and hormones associated with obesity (such as leptin, angiotensinogen, aldosterone stimulating factor, dipeptidyl peptidase 4, and resistin) that could explain the metabolic effect of adipose tissue on glomerular hyperfiltration were not measured. It is difficult to directly prove a causal relationship between abdominal fat distribution and glomerular hyperfiltration or renal dysfunction due to the cross-sectional design of this study. To demonstrate a causal relationship between abdominal fat tissue and glomerular hyperfiltration, the effect of abdominal fat tissue on kidney dysfunction should be investigated in prospective cohort settings.

This study shows that abdominal adipose tissue areas, especially subcutaneous adipose tissue area in men and visceral adipose tissue area in women, are associated with glomerular hyperfiltration, and the subcutaneous adipose tissue area in men is independently related with glomerular hyperfiltration, irrespective of body mass index. The risk of renal dysfunction in subjects with abdominal obesity—including subjects with a normal body mass index—should be further explored. Further research is needed to determine the mechanisms linking adipose tissue and altered renal hemodynamics. Additionally, prospective clinical studies investigating the effect of abdominal obesity and renal dysfunction should performed.
